# Unpacking the multimodal, multi‐scale data of the fast and slow lanes of the cardiac vagus through computational modelling

**DOI:** 10.1113/EP090865

**Published:** 2023-04-30

**Authors:** Michelle M. Gee, Eden Hornung, Suranjana Gupta, Adam J. H. Newton, Zixi (Jack) Cheng, William W. Lytton, Abraham M. Lenhoff, James S. Schwaber, Rajanikanth Vadigepalli

**Affiliations:** ^1^ Department of Chemical and Biomolecular Engineering University of Delaware Newark Delaware USA; ^2^ Department of Pathology and Genomic Medicine Daniel Baugh Institute of Functional Genomics/Computational Biology Thomas Jefferson University Philadelphia Pennsylvania USA; ^3^ Department of Physiology and Pharmacology SUNY Downstate Health Sciences University Brooklyn New York USA; ^4^ Burnett School of Biomedical Sciences, College of Medicine University of Central Florida Orlando Florida USA

**Keywords:** cardiovascular control, computational neuroscience, mathematical model, vagus nerve

## Abstract

The vagus nerve is a key mediator of brain–heart signaling, and its activity is necessary for cardiovascular health. Vagal outflow stems from the nucleus ambiguus, responsible primarily for fast, beat‐to‐beat regulation of heart rate and rhythm, and the dorsal motor nucleus of the vagus, responsible primarily for slow regulation of ventricular contractility. Due to the high‐dimensional and multimodal nature of the anatomical, molecular and physiological data on neural regulation of cardiac function, data‐derived mechanistic insights have proven elusive. Elucidating insights has been complicated further by the broad distribution of the data across heart, brain and peripheral nervous system circuits. Here we lay out an integrative framework based on computational modelling for combining these disparate and multi‐scale data on the two vagal control lanes of the cardiovascular system. Newly available molecular‐scale data, particularly single‐cell transcriptomic analyses, have augmented our understanding of the heterogeneous neuronal states underlying vagally mediated fast and slow regulation of cardiac physiology. Cellular‐scale computational models built from these data sets represent building blocks that can be combined using anatomical and neural circuit connectivity, neuronal electrophysiology, and organ/organismal‐scale physiology data to create multi‐system, multi‐scale models that enable in silico exploration of the fast versus slow lane vagal stimulation. The insights from the computational modelling and analyses will guide new experimental questions on the mechanisms regulating the fast and slow lanes of the cardiac vagus toward exploiting targeted vagal neuromodulatory activity to promote cardiovascular health.

## Abstract (highlights)

- **What is the topic of this review?**
  The vagus nerve is a crucial regulator of cardiovascular homeostasis, and its activity is linked to heart health. Vagal activity originates from two brainstem nuclei: the nucleus ambiguus (fast lane) and the dorsal motor nucleus of the vagus (slow lane), nicknamed for the time scales that they require to transmit signals.
- **What advances does it highlight?**
  Computational models are powerful tools for organizing multi‐scale, multimodal data on the fast and slow lanes in a physiologically meaningful way. A strategy is laid out for how these models can guide experiments aimed at harnessing the cardiovascular health benefits of differential activation of the fast and slow lanes.

## 1 INTRODUCTION

**FIGURE 1:**
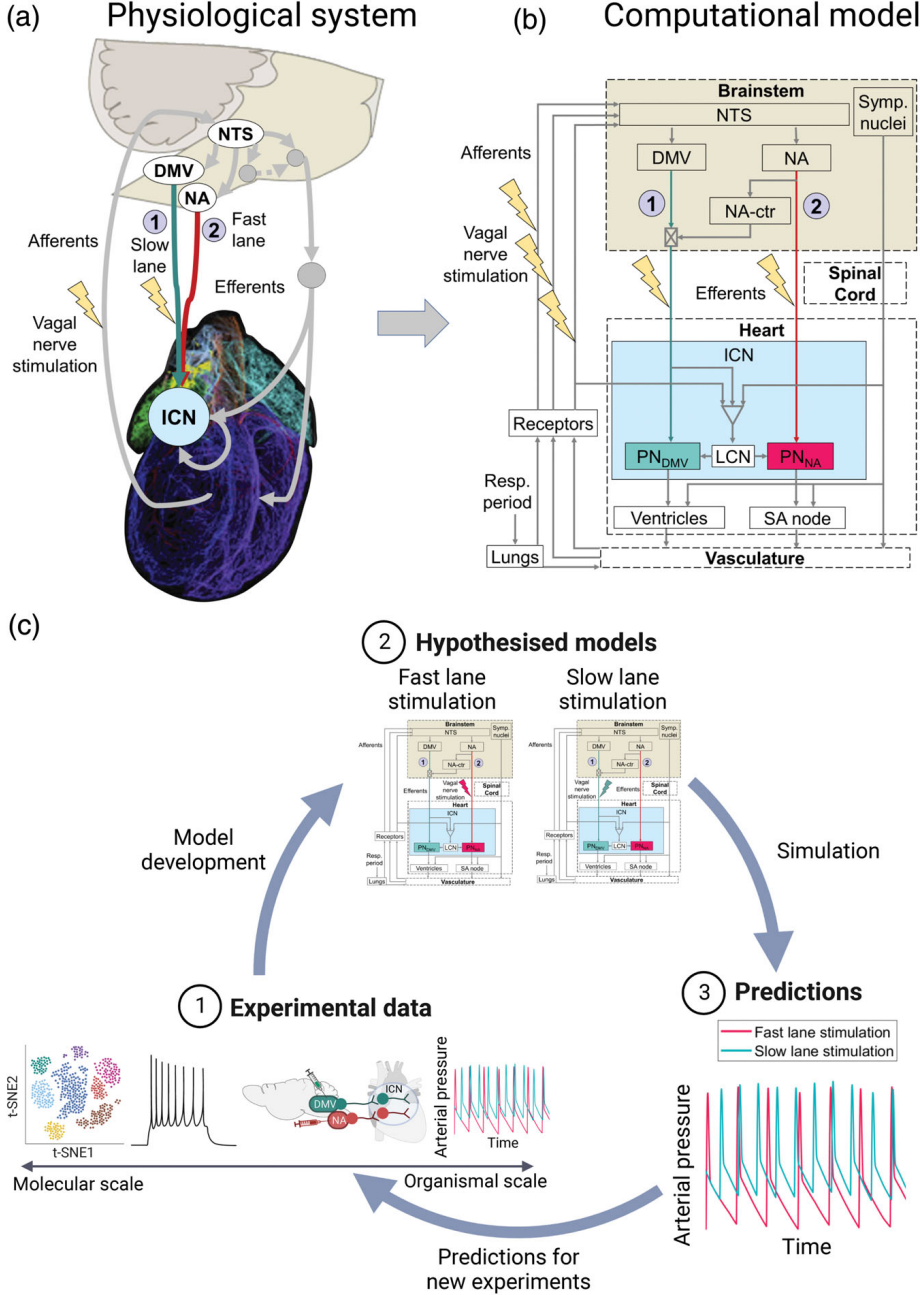
(a, b) Translation of the physiological control system regulating cardiovascular homeostasis via the fast and slow lanes of cardiovascular control (a) to a computational model (b). (c) The cycle of multi‐scale, multimodal data collection, development of models to represent hypotheses, and analysis of model predictions to generate new experimental hypotheses. We have combined multi‐scale data from single‐neuron transcriptomics, electrophysiology, neural tracing, and organism‐scale physiology into computational models representing selective fast and slow lane vagal stimulation. We could then use these models to simulate alternative scenarios of vagal nerve stimulation and compare the model predictions to experimental data to help frame questions for the next round of experiments. Lightning bolt symbols represent vagal nerve stimulation. DMV, dorsal motor nucleus of the vagus; ICN, intrinsic cardiac nervous system; LCN, local circuit neuron; NA, nucleus ambiguus; NA‐ctr, contractility contribution from the NA; NTS, nucleus tractus solitarius; PN, principal neuron; SA, sinoatrial. Figure created with BioRender.com.

The vagus nerve is a vital part of the body–brain axis that facilitates cardiovascular health and homeostasis in a closed‐loop feedback system (Figure 1a). The vagus contains both sensory afferents that transmit interoceptive signals to the nucleus tractus solitarius (NTS) and efferent motor outflow from the brainstem through the ‘fast’ and ‘slow’ lanes of the vagus to the heart's ‘little brain’, the intrinsic cardiac nervous system (ICN) (Armour, 2008). The ‘fast’ lane acts through myelinated B‐fibres originating from the nucleus ambiguus (NA) in the brainstem and is responsible for beat‐to‐beat cardiac regulation (Coote, 2013). The NA integrates baroreceptor and chemoreceptor inputs to produce reflex signals to the heart. The ‘slow’ cardiac regulation is produced by unmyelinated vagal C‐fibres originating from the dorsal motor nucleus of the vagus (DMV) and regulates ventricular contractility without substantial heart rate effects (Gourine et al., 2016).

Neural tracing experiments have revealed that the neural pathways via the NA and DMV remain distinct at the post‐ganglionic targets in the ICN, suggesting anatomically distinguishable ‘lanes’ of vagal control of cardiac physiology (Figure 1). The NA (fast) and the DMV (slow) were each injected with a different anterograde tracer (Cheng et al., 2004). Confocal imaging showed that the two lanes are anatomically distinct, as they innervate two distinct ICN neuron populations. The two lanes are also molecularly distinct given that the NA transmits signals to the ICN primarily via nicotinic receptors (Coote, 2013) and the DMV acts via muscarinic receptors (Mastitskaya et al., 2012).

Vagal activity is associated with cardiovascular health and protects the heart from ischaemic injury (Buchholz et al., 2018). Specifically, slow‐lane activity is one source of vagal cardioprotection and is blocked by the muscarinic receptor antagonist atropine (Buchholz et al., 2018; Mastitskaya et al., 2012). Restoring vagal tone using the bioelectronic treatment of vagus nerve stimulation (VNS) has shown promise (Buchholz et al., 2018). Experimental success led to clinical trials (De Ferrari et al., 2017), which in turn led to a new NIH Common Fund initiative, Stimulating Peripheral Activity to Relieve Conditions (SPARC), inspired partially by the compelling evidence that VNS could treat heart failure (Ardell et al., 2017).

Despite experimental evidence of the cardioprotective effects of VNS, a systems‐level understanding of cardioprotection remains elusive. Computational models of cardiovascular control have proven useful for organising and translating multi‐scale molecular, anatomical and physiological data into predictions of regulatory mechanisms that can guide new experimental questions (Gee et al., 2023; Kember et al., 2017; Park et al., 2020; Rogers et al., 2000; Ursino & Magosso, 2006; Vadigepalli et al., 2001). Now, multi‐scale and multimodal vagal data have been collected and organised in the publicly available SPARC portal (https://sparc.science). These new multimodal data, particularly single‐cell data, can be incorporated into multi‐scale computational models of cardiovascular control and physiology. We outline a control systems modelling framework that incorporates anatomical data on neural circuits, molecular data on neuronal functional states and physiological data from cellular‐scale to multi‐organ interactions to aid our understanding of vagal contributions to heart health.

## 2 AN ORGANISMAL‐SCALE FRAMEWORK FOR MODELLING THE FAST AND SLOW LANES OF THE CARDIAC VAGUS

Initial cardiac control models incorporated interdependent feedback loops of autonomic regulation (Hadaya & Ardell, 2020) through efferent sympathetic outflow and a single lane of vagal outflow (Kember et al., 2017; Ursino & Magosso, 2006; Vadigepalli et al., 2001). As models became more detailed, brainstem nuclei such as the NTS, NA and DMV were incorporated, establishing the two sources of vagal efferent tone (Park et al., 2020). However, Park et al. did not include a representation of the ICN, which contains separate populations of ICN neurons receiving inputs from the two lanes (Cheng et al., 2004). Kember et al. (2017) included a representation of the ICN as a network of neurons responsive to heart rate or blood flow demand that interacted with thoracic and central nervous system neurons. While this model was useful for exploring ICN adaptation following myocardial infarction, its structure did not account for the two lanes at the brainstem or ICN levels. These models did, however, establish a physiologically realistic modelling framework and paved the way for integrating data on the two lanes of vagal control.

Recent immunofluorescent staining data have suggested that the two anatomically distinct ICN neuron populations are also molecularly distinct based on their nicotinic and muscarinic receptor expression (Vadigepalli, 2022). Relatively uniform nicotinic receptor expression and varying muscarinic receptor expression were observed in rat ICN neurons. These data suggested that the DMV acts through muscarinic receptors and the NA acts through nicotinic receptors in the ICN, which is in agreement with physiological evidence (Coote, 2013; Mastitskaya et al., 2012). The varying ICN muscarinic receptor expression suggested that the ICN contains a neural subpopulation where the instantaneous dynamics of ionotropic nicotinic receptors dominate the acetylcholine response, and another neural subpopulation responsive on the slower metabotropic muscarinic receptor time scale.

We have built on the work by Park et al. by incorporating a representation of the ICN with two post‐ganglionic neuron populations, thus establishing the two lanes of vagal control (Gee et al., 2023). We accounted for the varying signal transmission time scales of the two lanes using a slower time constant for the muscarinic receptors compared to the nicotinic receptors. To tune the sigmoidal function parameters representing the two ICN populations, we used single‐cell electrophysiology recordings in response to VNS (Rajendran et al., 2019). We then used organismal‐scale data on heart rate and blood pressure responses to VNS to demonstrate that our two‐lane model could predict cardiovascular changes during VNS (Yamakawa et al., 2014). This top‐down modelling approach provides a framework for organising multimodal data to predict how the two vagal lanes contribute to cardiac regulation during VNS.

## 3 INCORPORATING SINGLE‐NEURON TRANSCRIPTOMIC DATA INTO MODELS OF THE FAST AND SLOW LANES OF THE CARDIAC VAGUS

Initial models of the cardiovascular control system and its components were useful for establishing modelling frameworks for incorporating electrophysiology and cardiovascular physiology data but relied on putative ion channels and neuromodulators (Rogers et al., 2000; Vadigepalli et al., 2001). In one such model, NTS ion channel combinations were used in a Hodgkin–Huxley formalism, which simulates voltage changes in neurons (Rogers et al., 2000). This single‐neuron model was used in a network model of NTS neurons that incorporated qualitative observations of NTS activity in response to vagal stimulus and arterial pressure increases. While this model was useful for exploring alternative inhibitory feedback structures among NTS neuronal populations involved in processing signals before transmitting them to the fast and slow lane brainstem nuclei, it was developed before the collection of single‐cell transcriptomics data sets that include specific ion channel combinations in NTS neurons (Park et al., 2014). Similarly, a previous cardiovascular control model included ICN neuron neuromodulation by dopamine and serotonin (Vadigepalli et al., 2001), but the extent of ICN neuromodulatory networks was largely unexplored before the single‐neuron transcriptomic mapping of the ICN (Moss et al., 2021).

**FIGURE 2:**
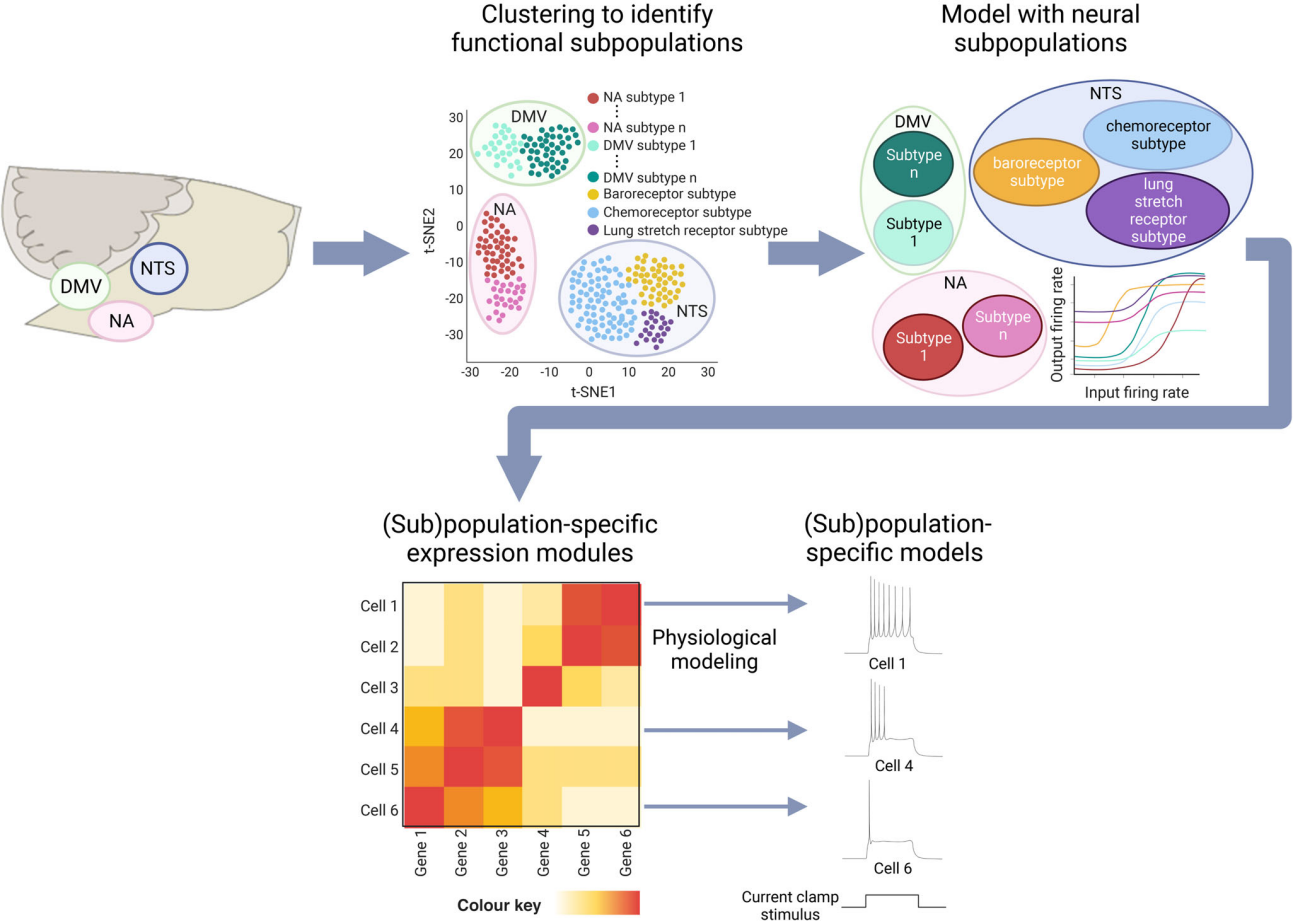
Uses of molecular‐scale, single‐cell transcriptomic data for developing physiological models with heterogeneous cell subpopulations. Neural subpopulations identified through clustering of single‐cell transcriptomic data are used to define the overall model components and structure. Each subpopulation is then unpacked and modelled at a finer‐grain scale using the single‐neuron transcriptomic data to define population‐specific expression modules. The expression modules contribute to a library of cellular models that are then used as building blocks to generate tissue‐scale models with heterogeneous cell populations. DMV, dorsal motor nucleus of the vagus; NA, nucleus ambiguus; NTS, nucleus tractus solitarius. Figure created with BioRender.com.

Recent analysis of single‐neuron transcriptomic data has revealed that the ICN is a heterogeneous neuronal population, with over 100 unique ion channel combinations identified from 405 sampled neurons (Moss et al., 2021). It is also a dynamic network driven by paracrine signaling of neurotransmitters and neuropeptides. In a bottom‐up modelling approach, ion channel information was mined from ICN transcriptomics data, and the respective Hodgkin–Huxley models were incorporated into single‐neuron models (Gupta et al., 2022). The ensemble of neuronal models was constrained using the ion channel combinations identified in the transcriptomics data (Figure 2). Due to heterogeneity in the single‐cell transcriptomic data, these models involve libraries of neuron types with unique ion channel combinations that display varying electrophysiological behaviour. These models can then be expanded to include representations of the combinations of neurotransmitters and neuropeptides expressed by each cell, also based on the transcriptomic data (Moss et al., 2021), thereby adding neuron–neuron paracrine signaling to the model and linking emergent network behaviour back to neuromodulatory mechanisms. We can then compare our models to ICN electrophysiology recordings which demonstrate diversity in cellular electrophysiological indicative of their heterogeneous transcriptomes (McAllen et al., 2011). These single‐neuron models can be combined into tissue‐scale networks of neurons by incorporating 3D spatial transcriptomic data to further constrain the neuron–neuron paracrine signaling (Moss et al., 2021).

Alternatively, or as a complementary step prior to the identification of single‐cell expression modules, high‐dimensional single‐neuron transcriptomic data are clustered to define neuronal subtypes. For example, single‐neuron NTS transcriptomic data (Park et al., 2014) indicated that cardiovascular‐related NTS neuronal subtypes are defined by their input types, such as baroreceptors (Figure 2). Thus, the NTS was modelled by neuronal subtypes corresponding to each input type (Park et al., 2020). Results from the model suggested that neuronal adaptation in the NA and DMV could compensate partially for systolic heart failure, while adaptation in the NTS could not. These results suggest that experimental questions on short‐term compensation for systolic heart failure should focus on neuromodulation of the NA and DMV.

## 4 INTEGRATING TRANSCRIPTOMICS‐BASED MODELS INTO ORGANISMAL‐SCALE MODELLING FRAMEWORKS OF THE FAST AND SLOW LANES OF THE CARDIAC VAGUS

**FIGURE 3:**
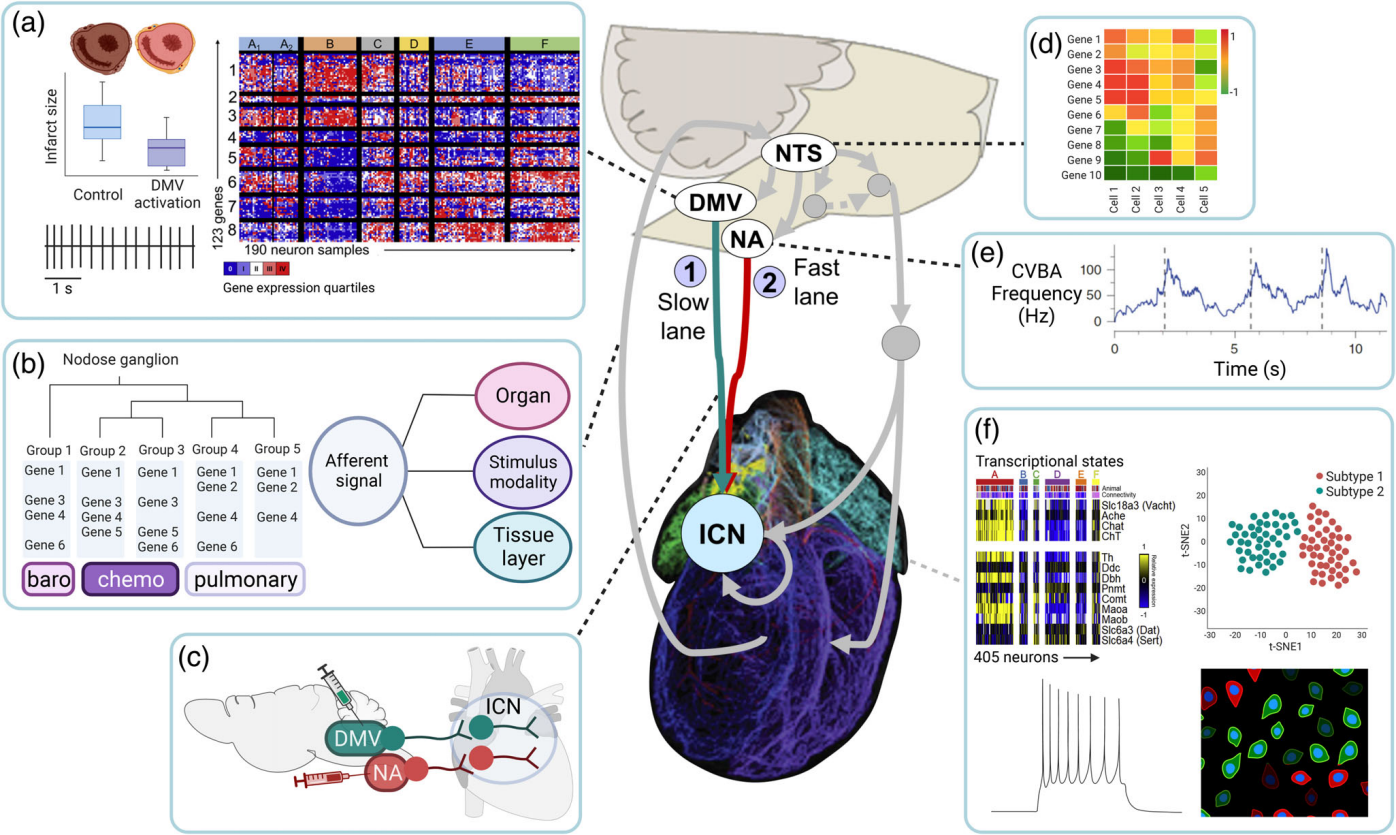
Multi‐scale, multimodal data sources for modelling two lanes of cardiovascular control. (a) DMV activation through optogenetics reduces infarct size following myocardial ischaemia (Mastitskaya et al., 2012). Single‐neuron transcriptomic analysis shows changes in transcriptional states in the DMV following the cardioprotective physiological manoeuvre remote ischaemic preconditioning (Gorky et al., 2021). Representative in vivo recording of a DMV neuron (Gourine et al., 2016). Image in top right panel adapted from Gorky et al. (2021). (b) Single‐cell transcriptomic analysis of vagal afferents in the nodose ganglion reveals cell subtypes based on sensory input (Kupari et al., 2019). Another analysis of vagal afferent data showed that neurons have a multidimensional coding architecture that contains information on the organ, the tissue layer and the stimulus modality of the sensory signal (Zhao et al., 2022). (c) Neural tracing indicates that the NA and DMV project to distinct populations of ICN neurons (Cheng et al., 2004). (d) Single‐neuron transcriptomic analysis of the NTS revealed that the neural subtype is driven by input signals from afferent neurons, leading to the classification of NTS subtypes by sensory input type in models (Park et al., 2014, 2020). (e) Electrophysiological data on the respiratory synchronised cardiac vagal branch activity (CVBA) from neurons in the NA (Farmer et al., 2016) has been used to incorporate cardio‐respiratory coupling into a model (Gee et al., 2023). The dashed vertical line indicates the start of post‐inspiration. Image adapted from Farmer et al. (2016). (f) Single‐neuron transcriptomic data of the ICN revealed a complex network of neurotransmitters and neuropeptides (Moss et al., 2021). With further transcriptomic analysis, we hope to find distinct populations of ICN neurons that differentially express nicotinic and muscarinic receptors, as has been observed with immunofluorescence staining data (Vadigepalli, 2022). These two phenotypes may be linked to the two observed ICN electrophysiological behaviours (McAllen et al., 2011). Image in top left panel adapted from Moss et al. (2021). DMV, dorsal motor nucleus of the vagus; ICN, intrinsic cardiac nervous system; NA, nucleus ambiguus; NTS, nucleus tractus solitarius. Figure created with BioRender.com.

Given our modelling strategy for incorporating single‐neuron transcriptomics data, we can now begin to populate our organismal‐scale modelling framework with single‐neuron models and neuronal subtypes to model the two vagal lanes and connect single‐cell transcriptomic data to organismal‐scale physiological function (Figure 3). We add single‐neuron models with known ion channels and neuromodulator combinations from single‐neuron transcriptomics data sets (Kupari et al., 2019; Zhao et al., 2022) to vagal afferents in the organismal‐scale model (Figure 3b). We used a similar approach to create an NTS model using single‐neuron transcriptomic data (Park et al., 2014) or we could analyse transcriptomic data to identify neural subtypes in the NTS and DMV (Figure 3a, d). We can combine these transcriptomic datasets with available brainstem and ICN electrophysiology datasets (Rajendran et al., 2019) to check the model predictions, in which we expect respiratory phase‐linked behaviour in NA neurons (Farmer et al., 2016) and tonic activity in DMV neurons (Gourine et al., 2016) (Figure 3e, f). To link brainstem and ICN populations, we use neural tracing data (Cheng et al., 2004) (Figure 3c). At an organismal scale, we use physiological data on heart rate and blood pressure changes in response to physiological perturbations such as VNS to validate the model predictions (Ardell et al., 2017; Buchholz et al., 2018).

These models provide a promising platform for in silico testing of targeted VNS of the fast versus slow lanes. The distinct nerve fibre types of the fast and slow lanes activate at different stimulus thresholds, meaning VNS can modulate the two lanes separately. A detailed quantitative accounting of the vagal afferent and efferent pathways in the closed‐loop model has the potential to enable in silico testing of the effect of shifting the VNS activation site. For example, cervical VNS would activate both afferents and efferents, whereas auricular VNS would primarily activate afferents. Differences in sensory information transmitted by cervical and auricular vagal afferent subsets may require a matching extension of the brainstem model components for appropriate downstream network processing. The model could be expanded to aid in the translation of animal data to human models (Brubaker & Lauffenburger, 2020) or to represent diseases such as heart failure (De Ferrari et al., 2017) that could be treated with VNS. Another promising avenue is to extend the modelling framework to study the effects of VNS on atrial fibrillation. Depending on the intensity of VNS, both pro‐ and anti‐arrhythmogenic effects of VNS have been reported (Kharbanda et al., 2022). The computational framework detailed here could be extended for evaluating the mechanisms and network interactions that govern the intensity‐dependent response to VNS in the context of intervention into atrial fibrillation. Populations of patient‐specific models could be developed and tuned using cardiovascular metrics to augment VNS clinical trial data, after following best practices for verification, validation and uncertainty quantification to demonstrate their credibility (Erdemir et al., 2020; Viceconti et al., 2021). Tools such as the SPARC portal provide systematic pipelines for mapping the experimental data used in model development to a common anatomical framework and provide streamlined access to data provenance for models on the portal (Osanlouy et al., 2021).

## AUTHOR CONTRIBUTIONS

Rajanikanth Vadigepalli: conception or design of the work. Michelle M. Gee, Eden Hornung, Suranjana Gupta, Adam J.H. Newton, Zixi (Jack) Cheng, William W. Lytton, Abraham M. Lenhoff, James S. Schwaber, Rajanikanth Vadigepalli: acquisition, analysis, or interpretation of data for the work. Michelle M. Gee, Eden Hornung, Suranjana Gupta, Adam J.H. Newton, Zixi (Jack) Cheng, William W. Lytton, Abraham M. Lenhoff, James S. Schwaber, Rajanikanth Vadigepalli: drafting of the work or revising it critically for important intellectual content. All authors have read and approved the final version of this manuscript and agree to be accountable for all aspects of the work in ensuring that questions related to the accuracy or integrity of any part of the work are appropriately investigated and resolved. All persons designated as authors qualify for authorship, and all those who qualify for authorship are listed.

## CONFLICT OF INTEREST

There are no conflicts of interest to report.
